# Attitudes and beliefs in Swedish midwives and obstetricians towards obesity and gestational weight management

**DOI:** 10.1186/s12884-020-03438-1

**Published:** 2020-12-03

**Authors:** Anne Christenson, Jarl Torgerson, Erik Hemmingsson

**Affiliations:** 1 Center for Obesity, Academic Specialist Center, Stockholm Health Services, Stockholm, Sweden; 2 Division of clinical epidemiology, Department of Medicine, Karolinska Institutet, Stockholm, Sweden; 3 Department of Molecular and Clinical Medicine, Institute of Medicine, Sahlgrenska Academy, University of Gothenburg, Gothenburg, Sweden; 4 The Swedish School of Sports and Health Sciences, Åstrand Laboratory of Work Physiology, Stockholm, Sweden

## Abstract

**Background:**

Gestational weight interventions are important in maternity care to counteract adverse pregnancy events. However, qualitative findings indicate potential obstacles in the implementation of interventions due to the sensitivity of the subject and existing obesity stigma. Pregnant women have reported disrespectful or unhelpful communication, while some midwives seem to avoid the topic, as not to upset women. This descriptive study aimed to provide knowledge about maternity care providers’ beliefs about obesity, and their attitudes towards gestational weight management.

**Method:**

A web survey was emailed to Swedish maternity care clinics. Existing questionnaires, “Beliefs About Obese People” (BAOP), “Perceived weight bias in health care” and “Attitudes toward obese patients” was used, supplemented with questions formulated for this study. An open free-text question allowed participants to provide a deeper and more nuanced picture of the topic.

**Results:**

274 respondents (75% midwives and 25% obstetricians) participated. One third of respondents found obesity to be a more sensitive topic than smoking or alcohol habits, and 17% of midwives agreed to the statement: “I sometimes avoid talking about weight so as not to make the pregnant woman worried or ashamed”. Having had training in motivational interviewing seemed positively associated with midwives’ inclination to talk about body weight, especially with women with obesity (*p* = .001), whereas years of working experience were not associated. Having received obesity education increased confidence in providing adequate information, but still only 46% felt they had enough knowledge to provide diet and exercise advice to pregnant women with obesity. Qualitative data revealed great empathy for women with obesity, and a wish to have more obesity education and access to other professionals.

**Conclusion:**

Swedish maternity care staff displayed empathy for women with obesity and found gestational weight interventions important, but almost one fifth of midwives sometimes avoid the subject of body weight for fear of upsetting women. Education about obesity facts, training in person-centered communication, i.e. motivational interviewing, and access to dieticians may facilitate gestational weight management implementation.

**Supplementary Information:**

The online version contains supplementary material available at 10.1186/s12884-020-03438-1.

## Background

Obesity in pregnancy and excessive gestational weight gain are associated with increased risks for several adverse events, such as gestational diabetes, pre-eclampsia, cesarean, intrauterine fetal death, as well as future obesity in the offspring [1]. These health risks are acknowledged in Swedish national recommendations, where the importance of qualified support during pregnancy, defined as person-centered advisory conversations, adapted to the woman’s specific age, health, and risk levels, is emphasized [2]. Guidelines propose that midwives should weigh women, discuss food habits and provide information about risks and recommendations regarding gestational weight gain [3, 4]. The recommendations presume that maternity care providers have extensive knowledge of the topic and sufficient training [2]*.*

Meanwhile, research findings describe several obstacles to the implementation of these recommendations. Qualitative findings indicate that some midwives perceive weighing and gestational weight management in women with obesity as difficult [5], more so than addressing, e.g. alcohol or tobacco use [6]. Health care providers in general have perceived communication with pregnant women with obesity to be challenging [7]. As a result, the topic of body weight is sometimes avoided for fear of inflicting shame or worries, especially in women with overweight or obesity [6].

Misconceptions about obesity are common, and prejudices and negative attitudes and behaviors towards individuals with obesity are evident [8]. The prevalence of stigmatizing attitudes is well documented among several populations [8], including medical settings [9, 10], and in the Swedish population in general [11]. Data is however lacking about attitudes towards obesity among midwives and obstetricians in Swedish maternity care.

Moreover, pregnant women with a BMI < 30 kg/m^2^ are less often the focus of weight interventions, even though about 40% of them gain excessively [12] in relation to the IOM guidelines, increasing the risk for adverse events and later obesity [13, 14].

To explore the presence of these possible obstacles, this study aimed to assess and describe Swedish midwives’ and obstetricians’ beliefs about obesity (I), their attitudes towards working with pregnant women with obesity (II), and their inclination to address the subject of gestational weight gain with women of different weight categories (III).

## Methods

This was a cross-sectional survey using a self-report questionnaire, with a predominantly quantitative design [15] supplemented with an open free-text question. Concurrent collection of quantitative and qualitative data can yield greater insight into complex phenomena than either approach alone [16, 17].

### Population and recruitment

During February and March 2019, Swedish midwives and obstetricians were recruited to complete an on-line web questionnaire. Participants’ emails were obtained by contacting the head of maternity care units from different parts of Sweden who in turn distributed an email with a link to the survey to their employees. Contact information was also obtained at midwifery meetings, where participants voluntarily provided their email for the web survey. Based on the total number of Swedish midwives (n ≈ 7000) and obstetricians (n ≈ 1600, we aimed to offer approximately 5% of them (*n* = 430) participation in the survey.

### Data collection

Data about attitudes, and how respondents perceive working with patients with obesity were assessed using an existing questionnaire created for a survey of advanced trainees in professional health disciplines [18]. The survey has been found to have adequate internal consistency as well as content- and structural validity [19]. For this study we used the parts “*Perceived weight bias in health care”,* (α = 0.63 to 0.89), and *“Attitudes toward obese patients”*, where participants were asked to indicate their level of agreement on a scale from 1 (strongly disagree) to 5 (strongly agree) (α = 0.70 to 0.83).

The *Beliefs about Obese Persons* (BAOP) questionnaire (α = 0.65 to 0.82) consists of eight statements about people with obesity, for example, *“most people with obesity eat more than non-obese people”* [20, 21]*. It uses* a 6-choice Likert rating scale ranging from − 3 (strongly disagree) to 3 (strongly agree). This questionnaire has been, and still is [22], widely used in many countries to assess different populations’ beliefs about people with obesity [11, 23, 24] and has been found to have adequate internal consistency and content validity [19].

To fully cover the research topics, new questions were formulated, see **Supplementary file** **1** (English version) and **Supplementary file** **2** (Swedish original). For content validity, they were based on results from our earlier interviews with midwives and pregnant women with obesity [6, 25], and from published data about common opinions and misconceptions about obesity [26]. The new questions were formulated as statements, where participants indicated their level of agreement on a 6-choice Likert scale from 1 (strongly disagree) to 6 (strongly agree). In two questions participants rated the importance of following the weight trajectory for women in different weight categories, (scores from 1 = not important at all to 6 = very important), and to what extent they discuss recommendations on gestational weight gain, (never = score 1 to always = score 5) with women in different weight categories (normal weight, overweight, obese).

Additionally, the questionnaire included an open-ended, free-text question with unlimited space for participants to provide any voluntary comments regarding both obesity and the survey itself.

### Translation procedure and validation of the questionnaire

Two independent researchers translated the English questionnaires (BAOP, “Attitudes towards obese patients”, and “Perceived weight bias in health care”) to Swedish. The Swedish versions were then compared and the wording were discussed until consensus was reached. A third researcher made a back-translation to English and then compared it with the original version, which resulted in minor revisions. Thereafter, the complete questionnaire was tested on ten persons within health care (including midwives) for content validity and user acceptability, and the final version was agreed upon in the research team.

Furthermore, as current recommendations suggest that the” person-first” approach should be used when talking about obesity, i.e.,” a person with obesity” instead of” an obese person” [27], the original questions were revised accordingly.

### Statistical analysis

Descriptive statistics were used to organize and summarize the characteristics of the population with help from IBM SPSS version 25. Chi-square test were used for analyzing between group differences.

### Qualitative data analysis

The qualitative data assessed from the additional voluntary comments were analyzed inductively with manifest content analysis [28]. All text material were reviewed and manually coded by AC and for researcher triangulation, discussed with JT until agreement [17]. Recurrent topics generated descriptive category headings, and subsequently coded statements were grouped under three categories. Quotes were chosen to illustrate the content of each category, as well as to increase credibility of the findings [29]. The quotes were translated from Swedish to English by the author and checked by two English-speaking peers.

## Results

### Response-rate

Out of 348 midwives and obstetricians who opened the emailed weblink, 79% (*n* = 274) replied to parts of the web survey, and 68% (*n* = 238) completed all questions. Among respondents, 75% (*n* = 205) were midwives, and 25% (*n* = 69) were obstetricians. In the analysis, all data were used, also from questionnaires that were only partially completed. Thus, the number of respondents varies between questions. Participants’ gender were not assessed, as less than 1% of midwives, and less than 30% of obstetricians in Sweden are men, and it was beyond the scope of this study to compare gender differences.

### Descriptive results

Table 1 shows the characteristics of participants who replied to parts, or all of the questionnaire. Participants were mainly middle-aged, with approximately 15 years of working experience in maternity care.

**Table 1:** Characteristics of participants

Characteristics	All (n = 274)	Midwives (*n* = 205)	Obstetricians (*n* = 69)
Age (y)^a^	49.7 ± 9.9 (28–70)	50.3 ± 10.0 (29–70)	47.7 ± 9.3 (28–65)
Years of working experience in maternity care (y)^a^	14.8 ± 10.0 (0.5–41)	14.4 ± 10.3 (0.5–41)	15.9 ± 9.1 (2–36)
Have received training in motivational interviewing	71%	76%	56%
Have received education on obesity in addition to their basic training	46%	45%	49%

^a^Data are presented as mean values, standard deviation and range

Table 2 displays respondents’ beliefs about obesity, and about women with obesity. The majority believed that women with obesity fear being judged based on their weight. Additionally, 48% of maternity care providers thought that women with obesity do not always tell what they really eat, and one third of respondents believed that pregnant women with obesity would prefer not to be weighed.

**Table 2:** Participants’ beliefs about pregnant women with obesity, and answers to Beliefs About Obese Persons questionnaire

Questionnaire items *Beliefs about pregnant women with obesity* ^a^	Median	Strongly/ moderately disagree	Slightly disagree	Slightly agree	Strongly/ moderately agree
Pregnant women with obesity					
fear being judged on the basis on their weight	5	3%	2%	36%	59%
don’t always tell what they really eat	4	3%	9%	40%	48%
are often unaware of the risks with obesity in pregnancy	4	9%	18%	29%	45%
know how to eat healthy	4	8%	23%	41%	28%
would prefer not to be weighed	4	12%	19%	36%	34%
have more psychological issues than other pregnant women	4	16%	33%	32%	19%
are often in need of professional psychological support	3	24%	40%	25%	12%
*BAOB* ^b^					
Obesity often occurs when eating is used as a form of compensation for lack of love or attention	−1	36%	39%	22%	4%
In many cases, obesity is the result of a biological disorder	−1	32%	38%	25%	4%
Obesity is usually caused by overeating	1	4%	6%	42%	47%
Most people with obesity cause their problem by not getting enough exercise	-1	26%	29%	36%	9%
Most people with obesity eat more than non-obese people	1	8%	15%	46%	32%
The majority of people with obesity have poor eating habits that lead to their obesity	2	4%	5%	39%	52%
Obesity is rarely caused by a lack of willpower	1	17%	26%	37%	20%
People can be addicted to food, just as others are addicted to drugs, and these people usually become obese	1	5%	15%	37%	43%

^a^*n* = 259 min =1, max = 6

^b^*n* = 246 min = −3, max = 3

Only 2% of respondents moderately or strongly agreed that talking about body weight may do more harm than good, and 77% considered it unprofessional not to weigh women and talk about risks with obesity even though it may be sensitive. One third of all participants thought that obesity was a more sensitive topic to talk about than smoking or alcohol habits. Of midwives, 17% (*n* = 31) slightly, moderately or strongly agreed to the statement: “I sometimes avoid talking about weight so as not to make the pregnant woman worried or ashamed”. Avoiding weight-related topics seemed more frequent (26%, *n* = 11) among midwives who had not received communication training, e.g., motivational interviewing (MI), compared to those who had (14%, *n* = 20). Among midwives with less than 5 years of working experience, only 56% (*n* = 43) had received MI training.

**Fig. 1:**
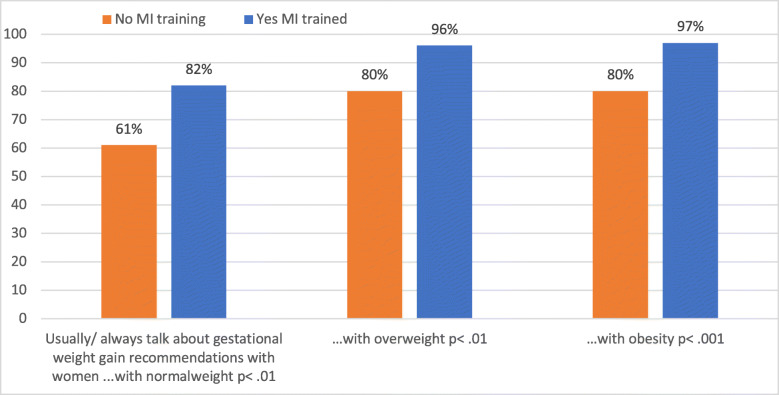
Midwives inclination to discuss body weight with women of different BMI-categories, depending on midwives training in motivational interviewing (of respondents to this question, n = 171 midwives were MI trained and n = 23 were not)

When comparing subgroups of midwives with, and without training in MI, the inclination to talk about body weight with women of different weight categories varied considerably (Fig. 1). Meanwhile, years of working experience was not significantly associated with tendency to avoid discussions about body weight, or with perceived need for education on obesity.

A majority of maternity care staff agreed that women with obesity could be difficult to deal with (Table 3), and among obstetricians, 31% prefer to treat non-obese women At the same time, 99% of all maternity care staff thought it was important to treat obese patients with compassion and respect, and only 3% claimed to dislike treating patients with obesity.

**Table 3:** Participants’ attitudes towards caring for patients with obesity

Questionnaire items	Median ^a^	Strongly/ moderately disagree	Both agree and disagree	Moderately/ strongly agree
I often feel frustrated with patients who have obesity	2.5	50%	40%	10%
Patients with obesity can be difficult to deal with	4	12%	31%	57%
I dislike treating patients with obesity	1	93%	4%	3%
I see no difference between patients with obesity and normal weight patients	3	34%	31%	35%
I feel confident that I provide quality care to patients with obesity	4	2%	22%	76%
I feel professionally prepared to effectively treat patients with obesity	3	19%	33%	48%
I would rather treat a non-obese patient than a patient with obesity	2	66%	19%	16%
I have heard other professionals in my field making jokes or negative comments about patients with obesity.	2	63%	15%	22%
My colleagues tend to have negative attitudes towards patients with obesity.	2	68%	20%	12%

^a^5-choice Likert scale. Min = 1, max = 5, *n* = 242

Data showed a trend of association between participants who had received education about obesity and those who had not, regarding perceived educational needs and confidence, see Table 4.

**Table 4:** Attitudes and opinions in participants with and without obesity education

Questionnaire item	Have received education on obesity	Have *not* received education on obesity	*p*-value
	Agrees with statement ^a^ % (n)		
With enough willpower anyone can lose weight	9 (11)	17 (23)	0.06
I need more education and knowledge about how to promote health in pregnant women with obesity	20 (24)	58 (77)	< 0.001
I have enough knowledge to give advice about diet and exercise to pregnant women with obesity	46 (55)	23 (31)	< 0.001
Exercise is better than diets for losing weight	22 (26)	31 (42)	0.08
I feel professionally prepared to effectively treat patients with obesity	52 (60)	44 (56)	0.16
Patients with obesity tend to be lazy	0	3 (4)	0.123
I feel that patients with obesity lack motivation to make lifestyle changes	15 (17)	21 (26)	0.22

^a^Agrees = responses of “moderately agree” or “strongly agree”

### Qualitative results

The open-ended question yielded data from 64 respondents (48 midwives and 16 obstetricians) which provided more depth and further illuminated the meaning of the quantitative answers. Statements were clustered into three categories (Table 5):

1. Reactions to the questions and survey topic: *“I find it hard to answer such categorical questions, as I do not see patients with obesity as a homogeneous group, but instead they are as diverse as normal weight patients”* Midwife no 12. *“It is an important topic, but at the same time it is hard to accomplish that much during pregnancy”* Obstetrician no 12.
2. Clarifications to quantitative answers:” *My reluctance to treat patients with a high BMI depends solely on that it is so much more difficult to do a good job,* e.g. *harder to perform an ultrasound, an operation, or to monitor the baby* etc. *and it is no fun to do a bad job.”* Obstetrician no 16.
3. Weight management improvements:” *I think maternity care should have access to a dietician who can see the overweight, and especially the obese patients. We midwives can support/motivate overweight women up to a certain point, but then something more is needed. Above all, I am a midwife, not a dietician”* Midwife no 4.

**Table 5:** Free-text answers presented in three categories, including category content description and illustrative quotes

Category I-III	Content description	Quotes
I. Reactions to the questions, and survey topic	Critical comments: Some respondents viewed the statements as condescending both to people with obesity or to health care staff, and others found the topic irrelevant, or questions difficult to answer. Some thought that body weight should not be brought up as it may make the pregnant woman unhappy.	*”This topic belongs to the 90’s. We have come further.”* Obstetrician no 11. *“I find it hard to answer such categorical questions, as I do not see patients with obesity as a homogeneous group, but instead they are as diverse as normal weight patients.”* Midwife no 6.
	Positive or confirmative comments: Respondents confirmed the relevance of the topic of obesity, and the sensitivity around weight and described how participants are working dedicatedly with this patient group.	*“It is a huge problem and I believe it is increasing. We now perceive normal weight patients as underweight.”* Midwife no 3, *“It feels like this questionnaire was a good reminder of how I relate to obese people and to myself. Thank you!”* Midwife no 39. *“We must strive towards a goal where obese patients are met with respect. It is deeply rooted among my colleagues, that obesity can be treated with diet and exercise.”* Obstetrician no 14 *“Since a pregnant woman is considered a risk -pregnancy, it is my obligation to make sure the pregnant women and her child will have an as safe pregnancy as possible. At the same time, I must consider that this may be a sensitive subject for the pregnant woman. It is a balancing act and it can be very difficult”.* Midwife no 57.
II. Clarifications to quantitative answers	Explanations to why respondents had answered that they found pregnant women with obesity difficult, or preferred treating non-obese women. Reasons provided were that higher perinatal risks and practical matters (e.g. more difficult ultrasounds, harder to operate on, and more oral glucose tolerance tests), rather than the personality of women with obesity affected respondents answers.	“*… and as obstetrician I am responsible for lives when a woman with severe obesity is giving birth. It is not because I have a cond*escendi*ng view on the person but because there is a factual medical risk.”* Obstetrician no 2. *“My reluctance to treat patients with a high BMI, depends solely on that it is so much more difficult to do a good job / … / because the patient’s BMI is associated with an incredible amount of medical risks”* Obstetrician no 16.
III. Suggested areas for weight management improvement	Stories from respondents own experiences reflected empathy and concern for women with obesity, as well as displayed areas where improvements of gestational weight gain management could be made. Factors mentioned were: lack of time, lack of access to dieticians, psychological support, or obesity treatment teams, a wish for written material about weight, access to water aerobics, walking groups, training in communication skills, and obesity education in general.	*”I think maternity care should have access to a dietician who can see the overweight, and especially the obese patients. We midwives can support/motivate overweight women up to a certain point, but then something more is needed. Above all, I am a midwife, not a dietician”* Midwife no 4 *“Most women know how they should eat, but because of anxiety or other hinders, they are unable to. Unless they have anorexia, there is no one we can refer them to who can take their eating problems seriously. This is a forgotten patient group, and we [midwives] lack education to help them. It feels rather stupid to just talk about the right diet, when there is so much more behind it.”* Midwife no 37 *“It is a challenge to talk about body weight with overweight women. However, It is not the knowledge I’m lacking, but the communication skills”* Midwife no 10. *“Give midwives more time to perform professional conversations. The medical duties take up 90% of the work time”* Midwife no 62.

## Discussion

In this sample of Swedish midwives and obstetricians, the importance of discussing and following the gestational weight trajectory in women with overweight or obesity was well recognized in over 90% of participants. However, results also showed that the topic of body weight was sometimes avoided due to the sensitivity of the subject, and that this avoidant behavior may occur more often among midwives who have not received training in motivational interviewing.

Though data should be interpreted with caution due to small numbers and a non-randomized selection of participants, these results strengthen the findings of earlier qualitative studies where some midwives perceived talking about obesity as such a sensitive subject that they occasionally avoided bringing it up [6, 30]. The trend in our data was that care providers who had received specific education on obesity, beyond their basic training, felt significantly more knowledgeable about obesity and able to provide diet and exercise advice compared to those without. Based on our data it seemed as if years of working experience did not automatically make up for the lack of specific education in this matter. In addition, even among those who had received specific obesity education beyond their basic training, less than half felt they had sufficient knowledge to give advice on diet and exercise, and one fifth of them expressed a need for more education to promote health in pregnant women with obesity.

Importantly, while some respondents agreed that they need more education on obesity, others explained that they possess the knowledge, but not the communication skills. Today, MI training is often part of the Swedish midwifery basic training curriculum. However, the extent and content vary between universities, and only about half of recently graduated midwives in our study claimed they had received training in MI. Also, even though 76% of these midwives had received MI training, studies using objective measures has shown that several bouts of practice and individualized supervision may be needed to achieve sufficient communication skills [5, 31].

Maternity health care providers in our study seemed aware that the topic of body weight may be sensitive or shameful for pregnant women with obesity, and avoiding the subject of body weight may sometimes be the correct approach. It is however important that a decision to refrain from bringing up the subject is based on the pregnant women’s best interest regarding medical and psycho-social needs, and not because the care provider lack confidence or sufficient knowledge.

Free-text answers such as: *“Above all, I am a midwife, not a dietician*” may indicate that aspects of weight management may be too much to add to midwives work load. This problem may not be solved by more education or time, but by involving other professionals. This is confirmed by earlier studies where midwives, as well as women with obesity, wish to have access to dieticians, counselors or to obesity clinics [6, 32].

Stronger beliefs about the individuals’ controllability of obesity have been associated with more stigmatizing attitudes [11]. Although non-significant, our data may indicate that participants, who had *not* received education on obesity, were more likely to agree with beliefs that are not scientifically grounded, such as: losing weight is only about having enough willpower. This is a persistent myth that may contribute to stigmatizing views, and not based on scientific findings [26].

Our findings may thereby add to earlier findings where education about obesity facts can not only increase confidence in clinicians to initiate weight discussions, but also decrease weight biased beliefs [33].

While more than half of respondents perceived women with obesity difficult to deal with, and one third of the obstetricians preferred to treat a non-obese patient, explanations were that it is practical matters, factual risks, and the extra work associated with obesity that generates this response, and not condescending views or stigmatizing attitudes. Our qualitative data revealed a substantial amount of empathy, concern and great awareness among respondents, of the difficulties that women with obesity struggle with. In addition, category 2, in Table 5, included answers that were written specifically to make sure that respondents’ quantitative answers would not be mistakenly interpreted as stigmatizing. Our results further showed that joking, and making derogatory comments about people with obesity happens also among maternity care providers (22% agreement), though it seems far less prevalent than in several other populations who have revealed 43–63% agreement [18].

### Strengths and limitations

A limitation of this study is the recruitment process. This was not a random sample, which decreases generalizability. Also, a reliable response rate could not be calculated as the link was mainly distributed via a second party, and thus we cannot know the exact number of email recipients. This obstacle was partly due to the new General Data Protection Regulation (GDPR) law from 2016 [34], which made heads of maternity care units unable to provide complete lists of staff or email-addresses. We therefore had to rely mainly on each head of department to distribute the link to relevant staff members. The new questions had not gone through a complete validation process. However, the items were based on interviews and had been pilot tested for content and construct validity and the new questions adds strength to the study as they fill a gap where optional questionnaires were lacking.

Socially more acceptable answers may be present in the survey as respondents may not want to reveal stigmatizing attitudes or admit lacking knowledge or skills to perform a work task [35]. However, self-reporting via an anonymous web survey might have facilitated honest self-disclosure. Furthermore, allowing respondents to explain themselves in the free-text answers, may have helped participants to present a more honest and nuanced picture.

## Conclusions

Swedish maternity health care providers in this study found gestational weight management for women with obesity highly important, but almost one fifth of midwives sometimes avoided the topic due to the sensitivity of the subject. In this sample, midwives who had received training in motivational interviewing seemed more inclined to address the subject of gestational weight gain recommendations with pregnant obese women, than midwives without MI-training. Less than half of maternity care providers claimed to have sufficient knowledge to give exercise and dietary advice to pregnant women with obesity.

Based on our results it could be argued that education about obesity facts, as well as training in communication skills offered to maternity care providers may be a way to improve weight management. Additionally, more time for lifestyle discussions, and access to other professionals, e.g. dieticians, or psychological support functions could potentially lessen midwives work burden.

## Supplementary Information

**Additional file 1: Supplementary file 1.** New questions developed for this study, English version.

**Additional file 2: Supplementary file 2.** New questions developed for this study, original version in Swedish.

## Data Availability

The data that support the findings of this study are available from the authors, but restrictions apply to the availability of these data, which were used under license for the current study, and so are not publicly available. Data are however available from the authors upon reasonable request and with permission of The Swedish Ethical Review Authority.

## Abbreviations

BMI: Body Mass Index
BAOP: Beliefs About Obese People
MI: Motivational Interviewing
IOM: Institute Of Medicine (US)
