# Supplementary material for: Attitudes and beliefs in Swedish midwives and obstetricians towards obesity and gestational weight management
Source: BMC Pregnancy Childbirth. 2020 Dec 3;20:755. doi: 10.1186/s12884-020-03438-1 (PMC7712607; doi:10.1186/s12884-020-03438-1)
Supplement: Supplementary file 1 — Additional file 1: Supplementary file 1. New questions developed for this study, English version. [file 12884_2020_3438_MOESM1_ESM.docx]

***Attitudes and beliefs towards obesity and gestational weight management Questionnaire***

***The following questions are developed specifically for this study***

**Here are some statements about obesity. Please mark the option which best matches your views.**

**Pregnant women with obesity…**

|  | **Strongly disagree** | **Moderately disagree** | **Slightly disagree** | **Slightly agree** | **Moderately agree** | **Strongly agree** |
| --- | --- | --- | --- | --- | --- | --- |
| …fear being judged based on their weight | 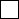 | 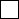 | 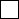 | 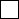 | 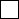 | 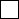 |
| …don’t always tell what they really eat | 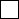 | 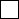 | 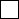 | 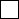 | 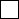 | 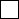 |
| …are often unaware of the risks with obesity in pregnancy | 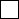 | 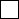 | 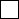 | 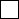 | 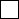 | 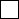 |
| …know how to eat healthy | 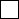 | 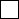 | 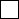 | 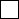 | 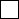 | 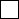 |
| …would prefer not to be weighed | 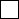 | 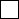 | 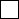 | 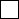 | 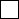 | 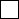 |
| …have more psychological issues than other pregnant women | 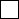 | 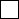 | 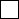 | 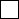 | 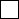 | 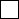 |
| …are often in need of professional psychological support | 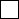 | 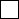 | 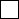 | 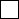 | 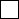 | 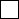 |

**Please mark the option that best matches your views.**

|  | **Strongly disagree** | **Moderately disagree** | **Slightly disagree** | **Slightly agree** | **Moderately agree** | **Strongly agree** |
| --- | --- | --- | --- | --- | --- | --- |
| Exercise is better than diets for losing weight | 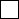 | 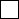 | 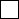 | 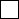 | 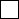 | 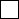 |
| With enough willpower anyone can lose weight | 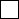 | 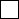 | 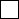 | 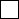 | 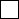 | 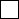 |
| I sometimes avoid talking about weight so as not to make the pregnant woman worried or ashamed | 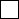 | 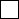 | 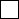 | 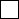 | 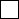 | 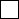 |
| I need more education and knowledge about how to promote health in pregnant women with obesity | 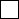 | 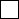 | 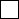 | 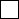 | 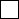 | 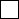 |
| Obesity is a more sensitive topic to talk about than smoking or alcohol habits | 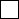 | 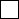 | 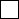 | 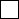 | 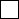 | 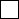 |
| It is unprofessional not to weigh women and talk about risks with obesity even though it may be sensitive | 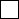 | 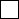 | 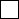 | 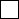 | 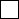 | 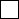 |
| Talking about body weight may do more harm than good | 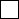 | 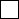 | 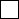 | 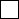 | 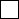 | 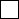 |
| I have enough knowledge to give advice about diet and exercise to pregnant women with obesity | 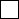 | 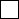 | 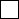 | 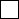 | 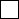 | 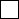 |

**How important do you consider it to be to follow the weight trajectory in a pregnant woman who is...**

|  | **Not important at all** | **Unimportant** | **Slightly unimportant** | **Fairly important** | **Important** | **Very important** |
| --- | --- | --- | --- | --- | --- | --- |
| ...normal weight? | 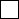 | 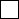 | 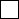 | 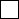 | 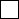 | 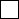 |
| ...overweight? | 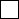 | 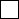 | 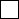 | 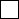 |  |  |
| ...has obesity? |  |  |  |  |  |  |

**To what extent do you bring up and discuss recommendations for gestational weigt gain with a woman who is…**

|  | **I never bring it up** | **rarely** | **sometimes** | **usually** | **I always bring it up** |
| --- | --- | --- | --- | --- | --- |
| ...normal weight? |  |  |  |  |  |
| ...overweight? |  |  |  |  |  |
| ...has obesity? |  |  |  |  |  |

**Is there anything more you would like to add regarding obesity, or about working with pregnant women with obesity? (free-text) If not, please klick NEXT to complete the survey!**

|  |
| --- |
|  |
