# Supplementary material for: Attitudes and beliefs in Swedish midwives and obstetricians towards obesity and gestational weight management
Source: BMC Pregnancy Childbirth. 2020 Dec 3;20:755. doi: 10.1186/s12884-020-03438-1 (PMC7712607; doi:10.1186/s12884-020-03438-1)
Supplement: Supplementary file 2 — Additional file 2: Supplementary file 2. New questions developed for this study, original version in Swedish. [file 12884_2020_3438_MOESM2_ESM.docx]

***Uppfattningar om fetma och viktinterventioner vid graviditet***

***Frågeformulär***

***Följande frågor är framtagna specifikt för denna studie.***

**Här följer några påståenden om fetma. Markera det alternativ du tycker stämmer bäst med din uppfattning.**

**Gravida kvinnor med fetma...**

|  | **Stämmer inte alls** | **Stämmer i låg grad** | **Stämmer ganska dåligt** | **Stämmer ganska bra** | **Stämmer i hög grad** | **Stämmer helt och hållet** |
| --- | --- | --- | --- | --- | --- | --- |
| ...är rädda för att bli bedömda utifrån sin vikt | 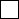 | 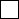 | 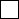 | 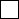 | 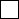 | 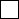 |
| ...berättar inte alltid vad de egentligen äter | 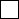 | 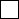 | 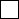 | 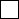 | 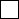 | 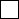 |
| ...känner ofta inte till riskerna med fetma vid graviditet | 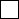 | 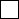 | 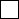 | 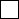 | 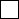 | 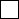 |
| ...vet hur man ska äta hälsosamt | 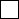 | 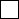 | 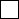 | 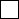 | 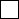 | 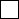 |
| ...vill helst slippa väga sig | 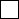 | 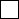 | 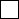 | 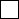 | 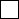 | 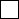 |
| ...har fler psykologiska problem än andra gravida kvinnor | 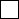 | 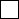 | 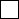 | 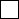 | 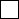 | 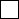 |
| ...behöver ofta professionellt psykologiskt stöd | 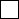 | 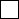 | 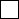 | 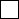 | 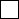 | 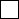 |

**Markera det alternativ du tycker stämmer bäst med din uppfattning.**

|  | **Stämmer inte alls** | **Stämmer i låg grad** | **Stämmer ganska dåligt** | **Stämmer ganska bra** | **Stämmer i hög grad** | **Stämmer helt och hållet** |
| --- | --- | --- | --- | --- | --- | --- |
| Motion är bättre än dieter för att gå ned i vikt | 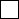 | 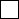 | 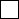 | 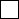 | 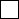 | 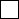 |
| Bara man vill tillräckligt mycket kan vem som helst gå ned i vikt | 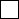 | 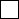 | 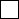 | 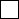 | 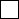 | 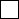 |
| Det händer att jag undviker att prata om vikten för att inte kränka eller oroa den gravida kvinnan | 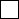 | 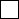 | 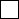 | 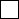 | 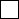 | 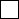 |
| Jag skulle behöva mer utbildning och kunskap om hur jag kan främja hälsa hos gravida kvinnor med fetma | 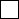 | 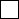 | 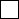 | 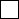 | 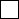 | 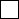 |
| Det är känsligare att prata om fetma än att prata om rökning eller alkoholvanor | 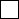 | 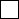 | 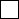 | 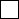 | 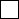 | 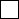 |
| Det är oprofessionellt att inte väga och prata om risker med fetma även om det är känsligt | 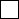 | 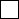 | 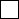 | 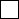 | 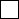 | 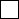 |
| Jag tror att det kan göra mer skada än nytta att prata om vikten under graviditet | 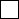 | 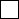 | 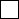 | 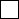 | 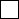 | 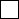 |
| Jag har tillräckliga kunskaper för att kunna ge råd om mat och motionsvanor till gravida kvinnor med fetma | 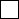 | 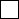 | 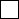 | 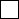 | 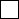 | 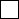 |

**Hur viktigt tycker du det är att följa viktutvecklingen hos en gravid kvinna som är...**

|  | **Inte viktigt alls** | **Oviktigt** | **Ganska oviktigt** | **Ganska viktigt** | **Viktigt** | **Mycket viktigt** |
| --- | --- | --- | --- | --- | --- | --- |
| ...normalviktig? | 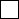 | 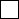 | 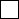 | 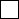 | 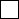 | 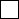 |
| ...överviktig? | 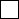 | 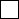 | 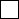 | 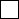 |  |  |
| ...har fetma? |  |  |  |  |  |  |

**I vilken utsträckning tar du upp och pratar om rekommenderad viktuppgång under graviditet med en kvinna som är...**

|  | **Jag aldrig upp ämnet** | **sällan** | **ibland** | **oftast** | **Jag tar upp det med alla** |
| --- | --- | --- | --- | --- | --- |
| ...normalviktig? |  |  |  |  |  |
| ...överviktig? |  |  |  |  |  |
| ...har fetma? |  |  |  |  |  |

**Är det något mer du vill nämna angående fetma eller om att arbeta med gravida kvinnor med fetma? (fritext) Annars klicka NÄSTA för att slutföra enkäten!**

|  |
| --- |
|  |
